# Phenotypic Characterization of Chicken Bursal Stromal Elements

**DOI:** 10.1155/1990/97618

**Published:** 1990

**Authors:** Richard L. Boyd, Trevor J. Wilson, Harry A. Ward, Kathy Mitrangas

**Affiliations:** 1 Department of Pathology and Immunology Monash Medical School Commercial Road Prahran Victoria 3181 Australia; 2 Department of Pathology and Immunology Monash University Medical School Prahran Victoria 3181 Australia

## Abstract

Many, if not all, of the different phases of intrabursal B-cell maturation are controlled by the stromal components. We have used an extensive panel of mAb to provide a detailed phenotypic profile of these cells. Antigenic specificities were defined for the entire surface epithelium, interfollicular surface epithelium, follicle-associated epithelium, basement membrane, basement membrane-associated epithelium. Several mAb were specific for the medulla, including those reactive with the stellate network of epithelial cells, isolated macrophages, and granular, apparently secreted antigens. One of these, MUI-92, appears to be bursa-specific. Two mAb reacted strongly with stellate cortical macrophages, one of which weakly stained similar cells in the medulla. MHC-class II antigens were expressed on endothelium of the corticomedullary junction, macrophagelike cells in the cortex, and medulla and B lymphocytes predominantly in the cortex. Collectively, these mAb have demonstrated the antigenically distinct nature of discrete regions in the bursa, but also the continuity of the surface epithelium with the corticomedullary junction and medulla. They represent excellent reagents for defining the stromal cell contribution to B-cell development.


*Developmental Immunology*, 1990, Vol. 1, pp. 41–51
Reprints available directly from the publisher
Photocopying permitted by license only

© 1990 Harwood Academic Publishers GmbH
Printed in the United Kingdom

### Phenotypic Characterization of Chicken Bursal Stromal Elements

RICHARD L. BOYD,* TREVOR J. WILSON, HARRY A. WARD and KATHY MITRANGAS

*Department of Pathology and Immunology, Monash University Medical School, Prahran, 3181, Victoria, Australia*

Many, if not all, of the different phases of intrabursal B-cell maturation are controlled by the stromal components. We have used an extensive panel of mAb to provide a detailed phenotypic profile of these cells. Antigenic specificities were defined for the entire surface epithelium, interfollicular surface epithelium, follicle-associated epithelium, basement membrane, basement membrane-associated epithelium. Several mAb were specific for the medulla, including those reactive with the stellate network of epithelial cells, isolated macrophages, and granular, apparently secreted antigens. One of these, MUI-92, appears to be bursa-specific. Two mAb reacted strongly with stellate cortical macrophages, one of which weakly stained similar cells in the medulla. MHC-class II antigens were expressed on endothelium of the corticomedullary junction, macrophagelike cells in the cortex, and medulla and B lymphocytes predominantly in the cortex. Collectively, these mAb have demonstrated the antigenically distinct nature of discrete regions in the bursa, but also the continuity of the surface epithelium with the corticomedullary junction and medulla. They represent excellent reagents for defining the stromal cell contribution to B-cell development.

KEYWORDS: Bursa, monoclonal antibodies, chicken, stroma, B cells, differentiation.

### INTRODUCTION

Despite the central importance of the avian bursa of Fabricius in formulating the dichotomy model of the immune system and the fact that this organ remains the only known centralized site for the generation of B lymphocytes, the exact mechanisms by which it functions are not known. As a primary lymphoid organ, the bursa appears to have the capacity to attract bloodborne precursors (Le Douarin, 1986), induce their commitment to the B-cell lineage (Weber, 1982; Boyd et al., 1983), stimulate extensive proliferation of B lymphocytes within the follicles (Pink et al., 1985) associated with which is the generation of antigen-receptor diversity probably through gene hyperconversion (Weill et al., 1986; Lassila et al., 1988), localize intestinal-derived antigens in the vicinity of developing B cells (Bockman and Cooper, 1973), and confer upon mature B lymphocytes the capacity to produce specific antibodies (Granfors et al., 1982). In addition, the bursa can function as a secondary lymphoid organ since histologically plasma cells are evident (Naukkarinen and Sorvari, 1982), and plaque-forming cells can be found following immunization (Van Alten and Meuwissen, 1972). In this regard, T cells have been identified near the bursa (Odend'hal and Breazile, 1980) and within individual follicles (Chen, Cooper, Wilson, and Boyd, unpublished observations). The bursa also does not function in isolation, but has receptors for steroids (Ylickomi et al., 1987) and can directly influence the development of the thymus (Droege, 1976). Clearly, the functional capacities of the bursa are very complex, and although it is widely accepted that the controlling elements are encompassed within the nonlymphocytic stromal components of this organ, their exact nature is poorly understood.

Histological and ultrastructural studies on the bursal stroma have demonstrated the interfollicular

*Corresponding author. Present address: Department of Pathology and Immunology, Monash Medical School, Commercial Road, Prahran, 3181, Victoria, Australia.

surface epithelial cells (IFE) to be relatively undifferentiated and continuous with those of the corticomedullary junction; these epithelial cells are supported by a basement membrane (Olah et al., 1975). The follicle-associated epithelial cells (FAE), which facilitate contact between medullary lymphocytes and lumenal contents (Sorvari et al., 1975), have specialized features, including a villous surface and a stratified network of stellate cells (Holbrook et al., 1974; Glick, 1983). During embryogenesis, the plical surface epithelium (SE) is infiltrated by mesenchymal "dark" cells, resulting in follicle development, the medullary component of which is supported by a stellate network of epithelial cells (Frazier, 1974; Boyd, Barr, et al., 1976) and dendritic reticulum cells, phagocytic macrophages (Naukkarinen and Sorvari, 1982) and "secretory" cells (Olah and Glick, 1978). The cortex is in contact with the vasculature and connective tissue, has only rare epithelial cells, but does have macrophages and "dark" reticular cells (Frazier, 1974; Glick, 1983).

A more refined approach we have used to defining stromal elements is the production of monoclonal antibodies to these cell types. This study represents an extension to our earlier work, which utilized polyvalent antisera to identify a reticulin-fiber framework specific for the bursal cortex (Boyd, Ward, et al., 1976), a bursa-dependent reticular-epithelial cell antigen(s) present in the bursa medulla and B-dependent areas in the spleen, particularly periellipsoidal sheaths (Boyd, Barr et al., 1976), and a gut-associated mucin present in the bursal SE and medulla (Boyd and Ward, 1984). Houssaint et al. (1986) described two mAb to bursal stromal antigens, BEP-1 reactive with the SE and basement membrane-associated epithelium (BMAE) and BEP-2 reactive with a cytoplasmic antigen of medullary and SE cells.

The present study confirms these findings and describes 15 additional bursal stromal-cell antigens. This panel of mAb has been very useful for identifying the ontogenic developmental relationship between the individual stromal components (Wilson and Boyd, 1990a) and together with comparative analysis of bursae from cyclophosphamide- or testosterone-treated chickens (Wilson and Boyd, 1990b), we have assigned tentative roles for these stromal cells in different phases of B-cell differentiation. Preliminary details of our mAb have been recently published (Boyd, Wilson, et al., 1987; Boyd, Mitrangas, et al., 1987).

### RESULTS

A panel of 31 mAb was selected, each of which was thoroughly characterized. It provided an extensive phenotypic profile of the bursal stromal components and clearly delineated discrete regions (Table 1). No two antibodies have identical specificities.

TABLE 1
Reactivity of mAb on Adult Chicken Bursa

| mAb (MUI-No.) | Localization[a] |
|---|---|
| 80 | SE |
| 51, 60 | Interfollicular SE; FAE-negative |
| 90 | BM |
| 58, 77 | BMAE[b] |
| 73 | BMAE; FAE; SE-negative |
| 59, 91 | SE; BMAE |
| 53, 70 | SE; BM |
| 55, 65 | SE; medulla-epithelial network |
| 54, 81 | SE; medulla-epithelial network<br>Cortex and medulla—fine granular staining |
| 62 | SE; medulla—keratin-negative cells, granules |
| 75 | SE-basal layer; medulla—epithelial network |
| 64, 74 | Medulla—epithelium |
| 92 | Medulla—outer epithelial cells |
| 67, 68, 69 | Medulla—keratin-negative cells (granules) |
| 72 | Cortex—stellate keratin-negative cells |
| 66 | Cortex—stellate keratin-negative cells<br>Medulla—stellate keratin-negative cells (weak) |
| 79 | Interfollicular macrophages |
| 78 (BL) | Cortex—lymphocytes<br>CMJ—endothelium<br>Medulla—isolated keratin-negative cells, some lymphocytes<br>SE—negative |
| 36 | Cortex—lymphocytes<br>Medulla—lymphocytes<br>CMJ—macrophagelike cells |

[a]Abbreviations: BM, basement membrane; BMAE, basement membrane-associated epithelium; CMJ, corticomedullary junction; FAE follicle-associated epithelium; SE, surface epithelium.
[b]Very weak staining of SE.

The complexity of the SE was revealed by at least nine distinct staining patterns. MUI-80 reacted with the entire SE (Fig. 1a), whereas MUI-51 stained only the interfollicular SE, being negative on the FAE (Fig. 1b). Phenotypic differences were further revealed within the multiple cell layers that form the SE and the similarities between the SE and medulla. MUI-90 was specific for the underlying basement membrane (Fig. 2a) and MUI-58 with the BMAE (Fig. 2b); whereas the latter was very weak to negative on the SE, MUI-91 strongly labeled both the SE and BMAE (Fig. 2c). MUI-77 was similar to MUI-58, but was distinguished by a different ontogenic expression (Wilson and Boyd, 1990a). MUI-73

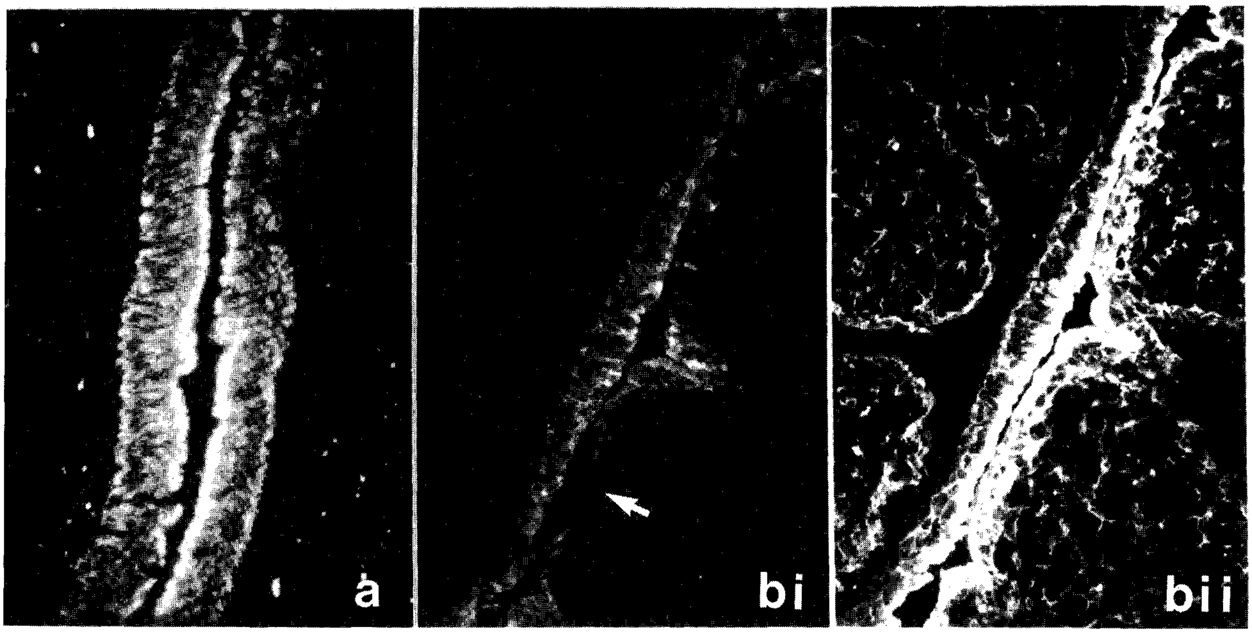

FIGURE 1. MUI-80 stained the entire bursal SE (a, ×400), whereas MUI-51 labeled only the interfollicular epithelium (b[i], ×125); note negative FAE (arrow). The double labeling of b(i) with antikeratin (×125) is shown in b(ii).

also stained the BMAE, but, in addition, stained the FAE and some areas of the medullary epithelium very weakly (Fig. 2d). MUI-53 and MUI-70 both stained the entire SE, but only stained the basement membrane or a single layer of epithelial cells at the corticomedullary junction (Fig. 2e). These antibodies were distinguished by their thymic reactivity; MUI-70 was specific for pan type 1 epithelium, which lines the subcapsule and perivascular regions, whereas MUI-53 only stained restricted regions of the subcapsular epithelium (Boyd, Wilson, et al., submitted).

Three mAb stained the SE and network of medullary epithelial cells, but each identified distinct determinants. MUI-75 stained the basal layer of the SE and the network of medullary epithelial cells (Fig. 3a). MUI-55 and MUI-65 reacted with the entire SE and stellate network of medullary epithelial cells; with the former, the staining was more diffuse, extensive, and appeared cytoplasmic (Fig. 3b), whereas the latter was more linear (Fig. 3c). MUI-62 also labeled the SE and medulla, but the cells in the medulla were keratin-negative and the staining very granular. MUI-67, 68, and 69 had similar medullary staining patterns to MUI-62, but were negative on the SE. These antibodies were distinguished by their ontogenic appearance (Wilson and Boyd, 1990a). MUI-69 was of particular interest because there was a marked heterogeneity between the follicles—some were strongly positive but others were weak or negative (Fig. 3d). This was not an artefact of the sections because serial sectioning through the follicles gave similar results. Whereas MUI-62 was reactive with thymic epithelial clusters and a mucin-like molecule throughout the respiratory and gastrointestinal tract, MUI-69 had very limited nonbursal reactivity, staining a subset of thymic medullary epithelial cell clusters and very infrequent goblet cells in the gastrointestinal tract.

MUI-92 was particularly interesting because it stained the outer medullary epithelial cells in the bursal follicles, but was negative on all other tissues tested (Fig. 3e).

In the bursa, three mAb were specific for keratin-negative macrophagelike cells. MUI-66 strongly stained isolated stellate macrophagelike cells in the cortex that were weaker and less frequent in the medulla (Fig. 4a). MUI-72 stained similar cells in the cortex, but was virtually negative on the medulla (Fig. 4b). The heterogeneity of macrophagelike cells was further exemplified by MUI-79, which stained such cells in the interstitial areas between follicles (Fig. 4c).

MUI-54 reacted with all bursal epithelium and, in this regard, closely resembled the panepithelium antikeratin reagent (Fig. 5a); the major dis-

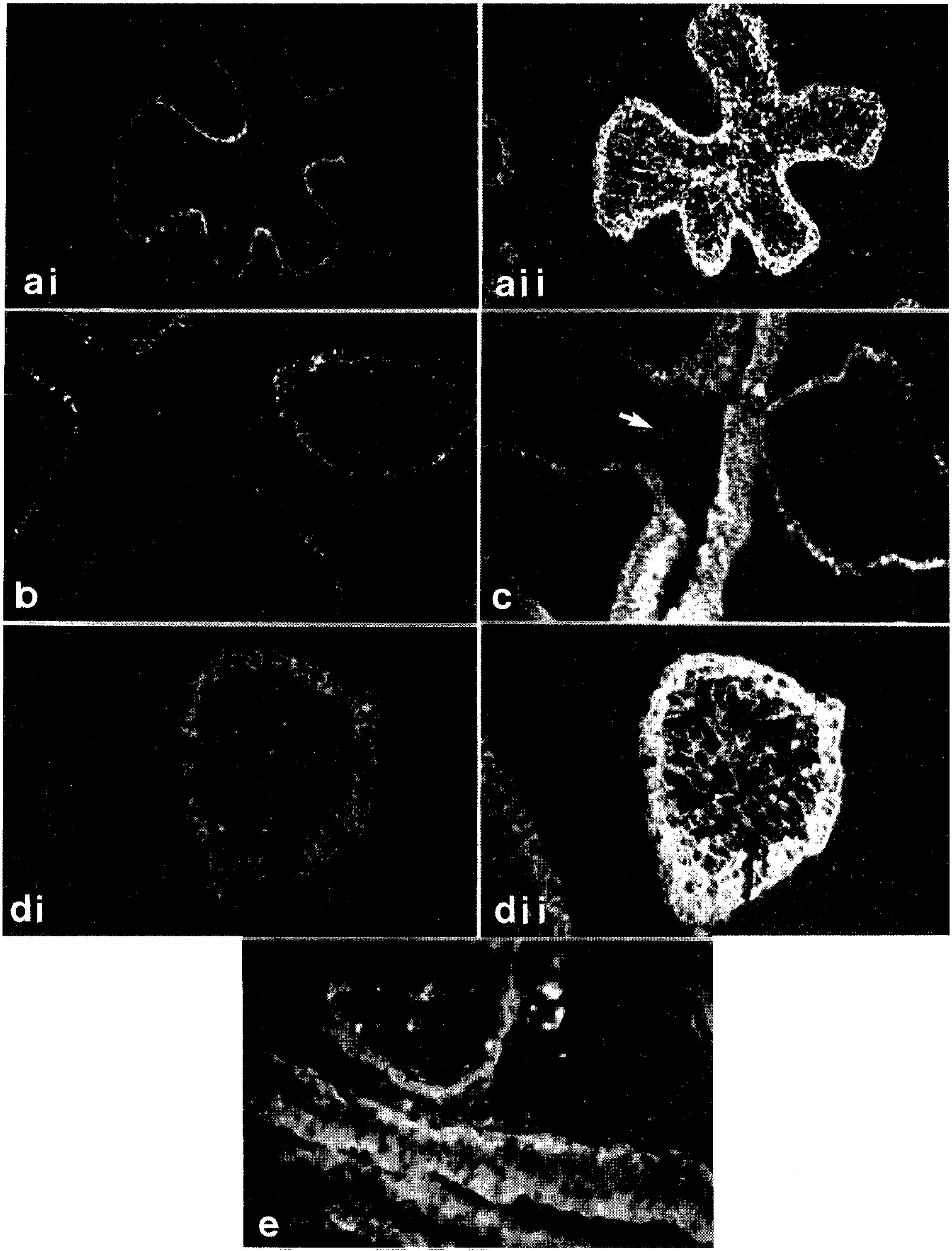

FIGURE 2. MUI-90 staining of the basement membrane delineating the corticomedullary junction (a[i], ×125). The double labeling of a(i) with antikeratin (×125) is shown in a(ii). Three mAb reacted with the BMAE at the corticomedullary junction: MUI-58 labeled only the BMAE (b, ×125), whereas MUI-91 stained the BMAE and the entire SE, excluding the FAE (arrow) (c, ×200), and MUI-73 the BMAE and weak areas of medullary epithelium (d[i], ×250). The double labeling of d(i) with antikeratin (×250) is shown in d(ii). MUI-53 stained the entire SE, but only the basement membrane or single layer of epithelial cells at the corticomedullary junction (e, ×400).

tinguishing feature was fine speckled staining throughout the cortex and medulla observed with the former. MUI-81 showed similar bursal reactivity, but in the thymus their patterns differed, with MUI-81 staining kerative negative cells not detected by MUI-54.

MUI-78, which reacts with BL (MHC class II; Boyd, Wilson, et al., 1987, submitted), stained predominantly lymphocytes and macrophages in the cortex, the endothelium at the corticomedullary junction, and isolated stellate cells in the medulla; there was no obvious staining of epithelium (Fig.

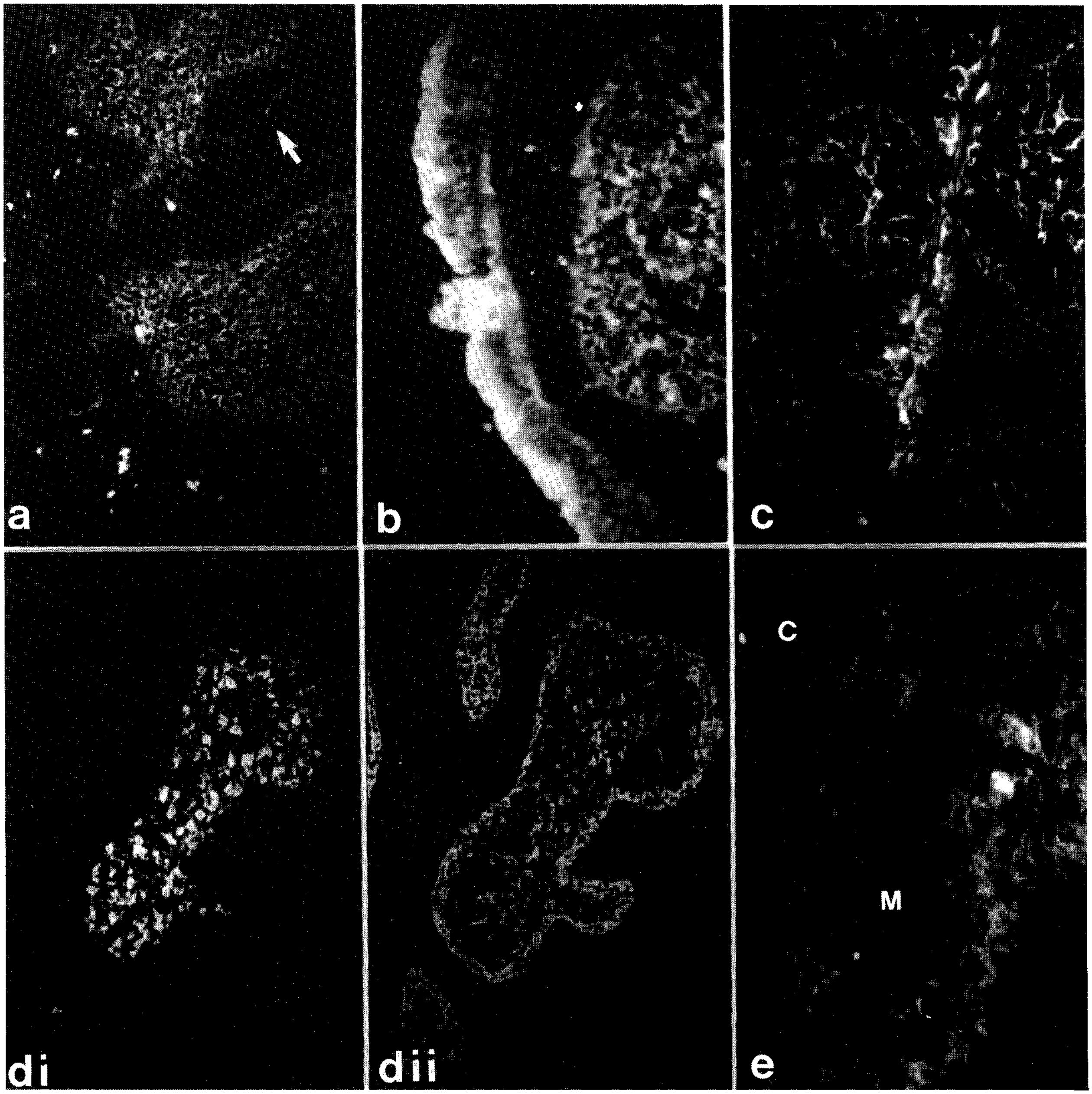

FIGURE 3. MUI-75 staining of the basal layer of the SE (arrow) and network of medullary epithelial cells (a, ×125). MUI-55 and MUI-65 both stained all layers of the SE in addition to the medullary epithelium; for the former, however, the staining was more diffuse and cytoplasmic (b, ×400), whereas the latter appeared restricted to plasma membrane (c, ×200). MUI-69 showed granular staining of keratin-negative cells in the medulla of follicles (d[i], ×125); the double labeling of d(i) with antikeratin is shown in d(ii) ( ×125). MUI-92 was bursa-specific and stained outer medullary (M) epithelial cells, the cortex (C) being negative (e, ×400).

5b(i)). MUI-36 is a pan-B-cell marker, reacting exclusively with all chicken B lymphocytes and a restricted subpopulation of macrophages at the corticomedullary junction and in the thymic medulla (Boyd, Mitrangas, et al., 1987).

All the MUI mAb were compared with antibodies specific for cytoskeletal components (Table 2). Antikeratin stained the entire SE, BMAE, and the stallate interconnecting network of medullary epithelial cells; the cortex was negative. Antiactin and antivimentin also reacted with medullary networks and the muscular layers; vimentin, unlike actin, was also present in the cortex. Stress fibers were only found in the muscle layer.

### Reactivity with Nonbursal Tissues

In the initial screening of the fusions, hybridomas showing broad tissue cross-reactivity were discarded. Many, however, reacted with the chicken thymus and these characteristics have been described elsewhere (Boyd, Wilson, et al., submitted). The nonlymphoid reactivities of the mAb were limited and have also been described elsewhere (Boyd, Wilson, et al., submitted).

TABLE 2
Distribution of Cytoskeletal Markers in Chicken Bursa

| Marker | Localization |
| --- | --- |
| Keratin | SE, BMAE, medulla—networks |
| Actin | Medulla—networks |
| | Smooth muscle layer |
| Vimentin | Cortex—isolated cells |
| | Medulla—networks |
| | Smooth muscle layer |
| Stress fibers | Smooth muscle layer |

Abbreviations: BMAE, basement membrane-associated epithelium; SE, surface epithelium.

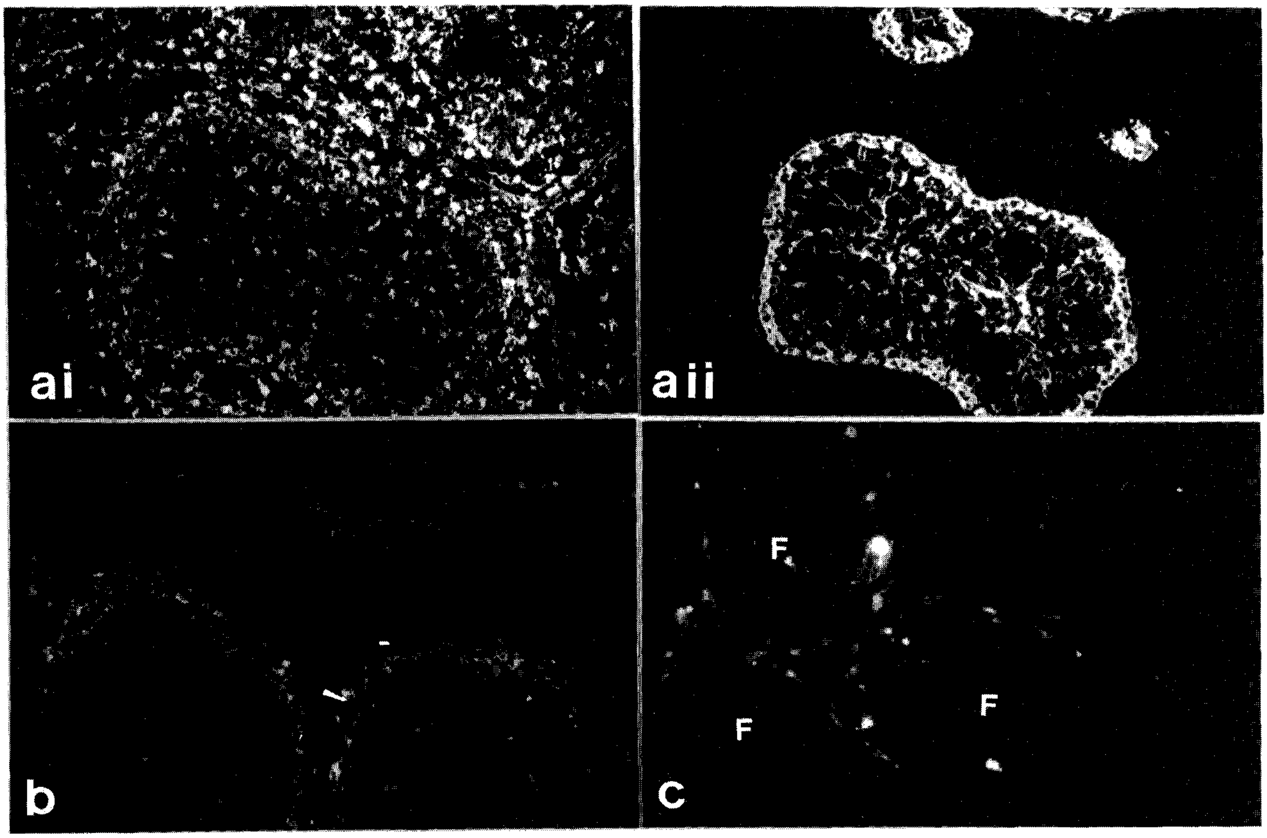

FIGURE 4. MUI-66 staining of stellate, keratin-negative cells predominantly in the cortex and less frequent in the medulla (a[i], ×125); the double labeling of a(i) with antikeratin is shown in a(ii) (×125). MUI-72 also stained stellate keratin-negative cells, but these were only found in the cortex (b, ×125). MUI-79 identified macrophages in the interstitial areas between follicles (F); the cortex and medulla were essentially negative (c, ×125).

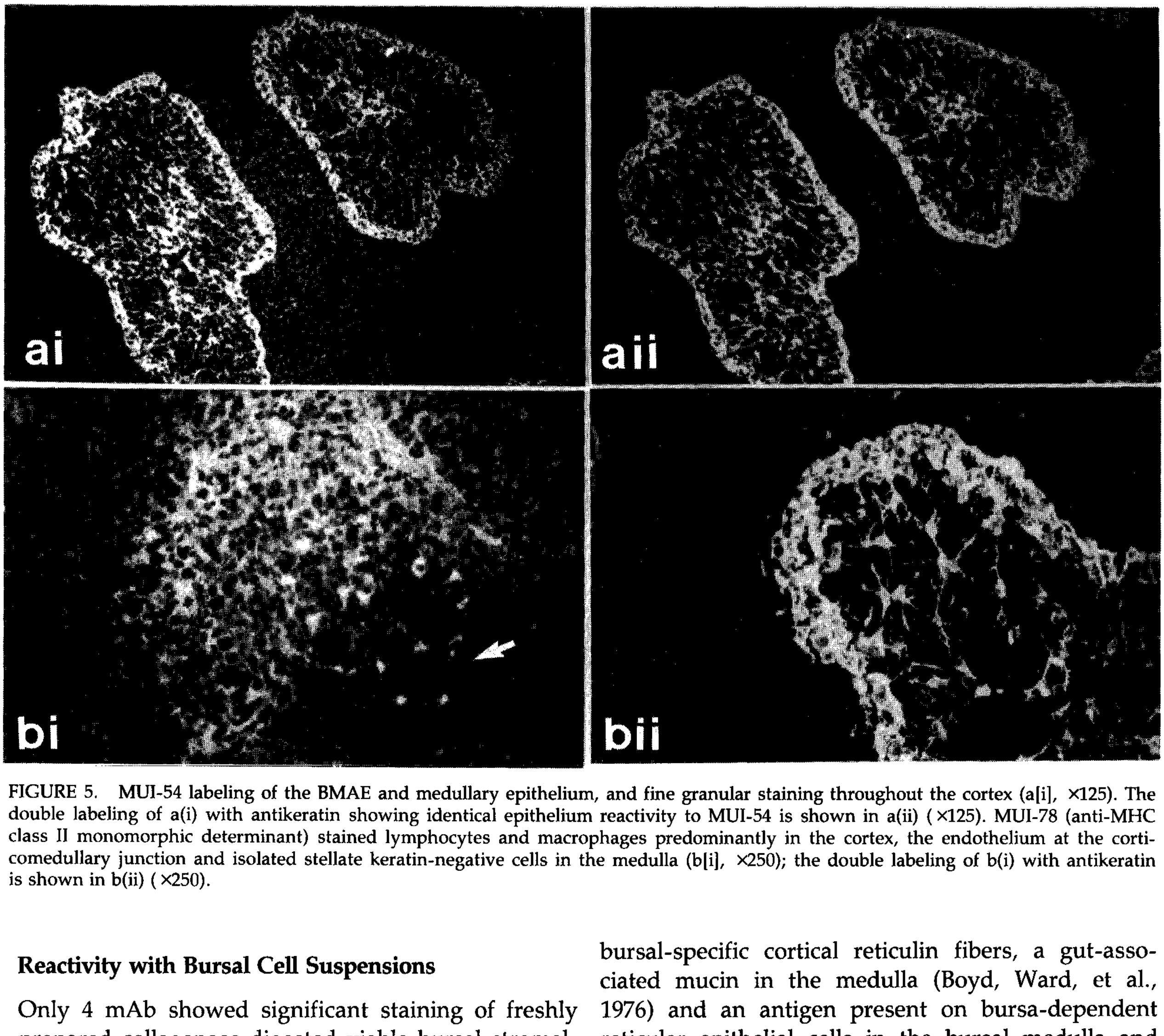

FIGURE 5. MUI-54 labeling of the BMAE and medullary epithelium, and fine granular staining throughout the cortex (a[i], ×125). The double labeling of a(i) with antikeratin showing identical epithelium reactivity to MUI-54 is shown in a(ii) ( ×125). MUI-78 (anti-MHC class II monomorphic determinant) stained lymphocytes and macrophages predominantly in the cortex, the endothelium at the corticomedullary junction and isolated stellate keratin-negative cells in the medulla (b[i], ×250); the double labeling of b(i) with antikeratin is shown in b(ii) ( ×250).

**Reactivity with Bursal Cell Suspensions**

Only 4 mAb showed significant staining of freshly prepared collagenase-digested viable bursal stromal-cell suspensions. MUI-61, 62, 36, and 78 stained approximately 14%, 30%, 60%, and 80%, respectively, of nonlymphocytic cells (based on 0° and 90° FACS scatter profiles) and also approximately 10%, 20%, 70%, and 60% of bursal lymphocytes, respectively.

### DISCUSSION

This study provides an extensive phenotypic profile of the stromal components of the bursa of Fabricius and demonstrates the complexity of the components that could potentially contribute to the microenvironment in this region. It complements our earlier data based on heteroantisera, which defined bursal-specific cortical reticulin fibers, a gut-associated mucin in the medulla (Boyd, Ward, et al., 1976) and an antigen present on bursa-dependent reticular epithelial cells in the bursal medulla and splenic germinal centers and periellipsoidal sheaths (Boyd and Ward, 1978). Recently, Houssaint et al. (1986) also described two mAb reactive with bursal stromal components: BEP-1, which stains mainly the SE and BMAE, and BEP-2, which binds to an intracytoplasmic antigen apparently secreted by medullary epithelial cells. In contrast to the present study, neither of these showed definite antigenic heterogeneity among the bursal epithelial cells, although the FAE were only weakly positive for BEP-1.

The present panel of mAb includes two reagents similar to BEP-1 and BEP-2, based on their staining patterns. These are MUI-91 and MUI-62, respectively. The remaining mAb clearly reveal epithelial cell heterogeneity. Whereas MUI-80 reacted exclusively with all the SE, MUI-51 stained only the interfollicular epithelium (non-FAE). This provides further proof for the specialized nature of the FAE, which have previously been shown to have ultrastructural and histochemical properties consistent with their ability to endocytose luminal contents with subsequent deposition in the underlying interconnecting network of reticular cells and fibers. A likely consequence of this is the presentation to differentiating B lymphocytes of intestinally derived antigens. In this context, the distribution of MHC class II antigens (MUI-78) in the bursa was interesting because the major medullary staining was of isolated stellate keratin-negative cells. Therefore, these may be functioning in an antigen-presenting capacity, the additional signals required for B-cell activation being provided by the often frequent T cells present in and around the follicles (Murthy et al., 1984). MHC class II antigens have been described on bursal lymphocytes (Ewert et al., 1984), which were shown here to be predominantly in the cortex, on the medullary epithelium of cyclophosphamide-treated bursae (Hoshi et al., 1988), on endothelium at the corticomedullary junction (Belo et al., 1985), and now on medullary stromal cells; however, their role in B-cell differentiation from multipotential precursors to functionally mature plasma cells remains obscure.

As distinct from BEP-1, MUI-55, and 65 reacted with the entire SE and the network of interconnecting medullary epithelial cells, including the basement-membrane associated epithelium. Since chick-quail chimera studies have demonstrated these regions to be endodermally derived (Le Douarin et al., 1976), these mAb may be valuable lineage markers. In addition to the distinction between the FAE and the interfollicular SE, heterogeneity was also revealed within the multiple layers that constitute the SE. MUI-90 reacted with the underlying basement membrane, but is unlikely to be directed against type IV collagen because of its lack of reactivity on intestinal and renal glomeruli-associated basement membranes and its species specificity (Boyd, Wilson, et al., submitted). Although the cellular origin of the individual components of basement membranes is still speculative, the contribution of the epithelial cells was indicated by the reactivity of MUI-53 and 70 with both the basement membrane and the entire SE. The distinctive nature of the BMAE was demonstrated by MUI-58 and 93, its continuity with the basal layer of the SE by MUI-91, and with the medullary epithelial network by MUI-54, 65, and 75. In view of the apparently undifferentiated nature of the BMAE revealed by transmission electronmicroscopy (Beezhold et al., 1983), it may be the precursor of both the outer SE and the stellate network of reticular epithelial cells forming the supporting framework of the medulla.

The complex nature of the medullary stroma was clearly revealed in this study. It consists of a network of stellate epithelial cells interspersed within which are isolated keratin-negative macrophagelike cells. It is not yet known if the four mAb reactive with the latter are detecting different populations of cells or different epitopes on the same cells. The apparent secretory nature of a subpopulation of medullary epithelial cells originally proposed by Olah and Glick (1978) was also demonstrated by MUI-69. This antibody was particularly interesting because it was negative on the SE and cortex, had very limited nonbursal reactivity, and showed marked variability in staining between different follicles. It would not be unreasonable to propose a specific function in B-cell maturation for this molecule and its variable intrabursal distribution raises the possibility of follicle heterogeneity. Whether this indicates different stages of B-cell maturation is unclear. BEP-2 of Houssaint et al. (1986) also reacts with an apparently secreted antigen in the medulla and SE, although no mention was made in that study of differences between follicles. MUI-62 resembles this mAb; we believe from ontogenic studies, however, that the antigen is secreted by the SE and taken up by the underlying medullary keratin-negative cells. MUI-92 is arguably the most important determinant in this study, because it is bursa-specific. Its restriction to outer medullary epithelial cells suggests a direct role in intrafollicular B-cell expansion.

In contrast to the complexity and specificity of the medullary stroma, only two mAb reacted preferentially with the cortex. MUI-72 extensively stained the stellate cells in the cortex, which would correspond to the reticular cells defined by electron microscopy (Olah et al., 1975). This apparently exclusive nature of the cortex and medulla provides further support for their different embryonic origins—the cortex being mesenchymal and the medulla endodermal (Le Douarin et al., 1975). However, whereas no endodermal (epithelial) cells were found in the cortex, the medulla did contain mesenchymal-derived macrophages. The relationship between the cortical and medullary B cells still remains very speculative. No differences in these two cell compartments were observed with

MUI-36, a pan-B-cell reagent. Similar results were found with an equivalent mAb (L22; Pink and Rijnbeek, 1983) and heteroantisera to B lymphocytes, although a fetal-associated antigen, CFAA, was found on cortical but not medullary B cells and conversely, a mature B-cell marker, CMBLA, was found only in the medulla (Boyd and Ward, 1984). This suggests that the medulla contains more mature B lymphocytes, but further study on the lineage relationships between these areas is required.

Several of the mAb described have reacted with macrophagelike cells. In addition to MUI-72 on cortical reticular cells, MUI-79 reacted with macrophages in the interfollicular regions, MUI-36 with those at the corticomedullary junction (and thymic medulla, Ward et al., in preparation), MUI-66 with those found extensively in the cortex and less abundantly in the medulla, and MUI-78 (MHC class II) with a subpopulation within the medulla. MUI-66 and 79 also react with blood monocytes and macrophages throughout many tissues (Boyd and Wilson, unpublished observations). None of these reagents are truly pan-macrophage in comparison to the recently described mAb ChNL-681 (Jeurissen et al., 1988), which has more general reactivity.

In summary, this panel of mAb reveals the marked heterogeneity of the bursal stroma and facilitates detailed studies on the nature of the microenvironment of this organ. Two approaches that have been very valuable for assigning a functional role of the cells involved to distinct phases of intrabursal B lymphocyte development have been to examine their ontogenic development and their status in chickens treated with cyclophosphamide as compared to testosterone, which ablates the functional microenvironment. The results of former studies are presented in an accompanying paper and the latter are in press (Wilson and Boyd, 1990b).

### MATERIALS AND METHODS

### Chickens

Australorp ×White Leghorn F1 hybrid chickens (4–8 weeks old) obtained from Research Poultry Farm (Research, Victoria) were used throughout this study.

### Stromal-Cell Preparation

Bursae and thymuses were extensively teased apart in serum-free RPMI 1640 at 4°C and the freed lymphocytes discarded. The remaining tissue aggregates and follicles were digested to a single cell suspension by incubation in an enzyme solution consisting of collagenase type IV (0.15%), trypsin (0.15%), and DNase (0.01%) (all from Boehringer-Mannheim). For immunization or antigenic analysis of stromal cells, trypsin was omitted. The stromal cells were then collected by repeated sedimentation through newborn calf serum at 1 g for 30 min, 4°C.

### Production of mAb

The experimental details for production of the mAb follow standard protocols and have been published elsewhere (Boyd, Wilson et al., 1987). Briefly, BALB/c mice were immunized i.p. with 0.2 ml serum-free RPMI 1640 containing 100 μl of packed stromal-cell-rich preparations, at weekly intervals. Three days after the third injection, the spleen cells were fused with log growth phase P3/NS-1/1-Ag/4-1 (NS-1) cells, HAT added on day 1 and HT at day 7. Hybridoma supernatants were initially screened by indirect immunofluorescence on 4 μm frozen sections of composite blocks of snap-frozen thymus, bursa, and spleen; those showing broad tissue cross-reactivity were discarded. Selected hybridomas were cloned at least twice by limiting dilution.

For indirect immunofluorescent staining, sections were completely covered in supernatant, incubated for 20 min at room temperature, washed three times (1×1 min, 2×5 min) with PBS (gentle shaking), and reincubated with conjugate for a further 20 min, and washed as before. Control preparations were treated with NS-1 conditioned medium. The conjugate was an FITC-labeled affinity purified $F(ab')_2$ sheep anti-mouse Ig (final dilution, 1:100, DDAF, Silenus Labs, Melbourne). Sections were mounted in veronal buffered glycerol or Permafluor (Lipshaw, Detroit), and examined with a Zeiss Axioskop microscope and photographed with a Zeiss MC100 automatic camera and Ektachrome ASA 1600 film.

### Specificity Analysis

All mAb were tested on cryostat sections of bursa, thymus, spleen, kidney, liver, lung, glandular stomach, small intestine, brain, heart, and skin. As an indication of species-specificity, the mAb were tested on sections of rabbit liver, mouse kidney, rat stomach, and cultured 3T3 fibroblasts. Reactivity with epithelial cells was determined by double labeling with a rabbit antikeratin (broad spectrum, dilution 1:200; Dako, Santa Barbara, California) and developed with a rhodamine conjugated goat-anti-rabbit Ig (dilution 1:50, Silenus Labs).

To determine reactivity of the mAb with plasma membrane determinants, immunofluorescence analysis was performed on fresh bursal stromal-cell suspensions. The cells (50 $\mu l$) were incubated with mAb (100 $\mu l$) for 20 min, 4°C, washed twice in 5 ml RPMI 1640 containing 10% newborn bovine serum (150 g, 5 min), and incubated with 50 $\mu l$ of the FITC-antimouse conjugate (final dilution, 1:100) for a further 20 min, 4°C. The cells were again washed twice and 25-$\mu l$ aliquots transferred to multiwell glass slides (Flow Labs). They were then rapidly air dried, fixed in absolute ethanol for 30 seconds, dried and washed in PBS for 10 min prior to mounting for immunofluorescence analysis.

### ACKNOWLEDGMENTS

This work was supported by grants from the National Health and Medical Research Council of Australia, the Anti-Cancer Council of Victoria and Monash University Special Research Grants.

*(Accepted January 18, 1990)*

### REFERENCES

Beezhold D.H., Sachs H.G., and Van Alten P.J. (1983). The development of transport activity by embryonic follicle-associated epithelium. J. Retic. Soc. **34:** 143–152.

Belo M., Martin C., Corbel C., and Le Douarin N.M. (1985). A novel method to bursectomize avian embryos and obtain quail-chick bursal chimeras. Immunocytochemical analysis of such chimeras by using species-specific monoclonal antibodies. J. Immunol. **135:** 3785–3794.

Bockman D.E., and Cooper M.A. (1973). Pinocytosis by epithelium associated with lymphoid follicles in the bursa of Fabricius, appendix and Peyers patches. An electron microscopic study. Am. J. Anat. **136:** 455–478.

Boyd R.L., and Ward H.A. (1978). Lymphoid antigenic determinants of the chicken: Cellular representation and tissue localization. Immunology **34:** 9–17.

Boyd R.L., and Ward H.A. (1984). Lymphoid antigenic determinants of the chicken: ontogeny of bursa-dependent lymphoid tissue. Dev. Comp. Immunol. **8:** 148–167.

Boyd R.L., Barr I.G., Ward H.A., and Muller H.K. (1976). Antigenic and functional properties of bursal and thymic reticular epithelial cells. Folia. Biol. **25:** 310.

Boyd R.L., Mitrangas K., Ramm H.C., Wilson T.J., Fahey K.J., and Ward H.A. (1987). Chicken B lymphocyte differentiation: ontogeny, bursal microenvironment and effect of IBD virus. Prog. Clin. Biol. Res. **238:** 41–51.

Boyd R.L., Ward H.A., and Muller H.K. (1976). Antiserum specific for reticulin fibres of the bursa of Fabricius. Intl. Arch. Allergy Appl. Immunol. **50:** 129–132.

Boyd R.L., Ward H.A., and Muller H.K. (1983). Bursal and thymic epithelial cells in the chicken: induction of B- and T-lymphocyte differentiation by in vitro monolayer cultures. J. Retic. Soc. **34:** 383–393.

Boyd R.L., Wilson T.J., Mitrangas K., and Ward H.A. (1987). Characterization of chicken thymic and bursal stromal cells. Prog. Clin. Biol. Res. **238:** 29–39.

Boyd R.L., Wilson T.J., van de Water J., Haapanen L.A., Ward H.A., and Gershwin M.E. (Submitted). Phenotypic analysis of avian thymic stroma: complexity of the microenvironment and abnormalities associated with avian scleroderma, an inherited fibrotic disease of line 200 chickens.

Droege W. (1976). The antigen-inexperienced thymic suppressor cells: a class of lymphocytes in the young chicken thymus that inhibits antibody production and cell-mediated immune responses. Eur. J. Immunol. **6:** 279–287.

Ewert D.L., Menchus M.S., Chen C.-L.H., and Cooper M.D. (1984). Analysis of structural properties and cellular distribution of avian Ia antigen by using monoclonal antibody to monomorphic determinants. J. Immunol. **132:** 2524–2530.

Frazier J.A. (1974). The ultrastructure of the lymphoid follicles of the chicken bursa of Fabricius. Acta. Anat. **88:** 385–397.

Glick B. (1983). Bursa of Fabricius. In: Avian Biology VII, Farner D.S., King J.R., and Parkes K.C. Eds. (New York: Academic Press), pp. 443–500.

Granfors K., Martin C., Lassila O., Suvitaival R., and Toivanen P. (1982). Immune capacity of the chicken bursectomized at 60 hr of incubation. Production of immunoglobulin and specific antibody. Clin. Immunol. Immunopathol. **23:** 459–469.

Holbrook K.A., Perkins W.D., and Glick B. (1974). The fine structures of the bursa of Fabricius: "B" cell surface configuration and lymphoepithelial organization as revealed by scanning and transmission electron microscopy. J. Retic. Soc. **5:** 300–311.

Hoshi S., Nunoya T., and Ueda S. (1988). Identification of B-L antigens on reticular epithelial cells of the bursa of Fabricius. Microbiol. Immunol. **32:** 173–186.

Houssaint E., Diez E., and Hallet M. (1986). The bursal microenvironment: phenotypic characterization of the epithelial component of the bursa of Fabricius with the use of monoclonal antibodies. Immunol. **58:** 43–49.

Jeurissen S.H.M., Janse E.M., Koch G., and de Boer G.F. (1988). The monoclonal antibody CVI-CHNL-681 recognizes cells of the monocyte-macrophage lineage in chickens. Dev. Comp. Immunol. **12:** 855–864.

Lassila O., Alanen A., Lefkovits I., Cooper M.D., and Pink J.R.L. (1988). Immunoglobulin diversification in embryonic chicken bursae and in individual bursal follicles. Eur. J. Immunol. **18:** 943–949.

Le Douarin N.M. (1986). The microenvironment of T and B lymphocyte differentiation in avian embryos. Cur. Top. Dev. Biol. **20:** 291–313.

Le Douarin N.M., Houssaint E., Jotereau F.V., and Belo M. (1975). Origin of haemopoietic stem cells in embryonic bursa of Fabricius and bone marrow studied through interspecific chimeras. Proc. Natl. Acad. Sci. USA **72:** 2701–2705.

Murthy K.K., Odend'hal S., and Ragland W.L. (1984). Demonstration of T lymphocytes in the bursa of Fabricius of the chicken following cyclophosphamide treatment. Dev. Comp. Immunol. **8:** 213–218.

Naukkarinen A., and Sorvari T.E. (1982). Morphological and histochemical characterization of the medullary cells in the bursal follicles of the chicken. Acta. Path. Microbiol. Immunol. Scand. Sect. C **90:** 193–199.

Odend'hal S., and Breazile J.E. (1980). An area of T cell localization in the cloacal bursa of white leghorn chickens. Am. J. Vet. Res. **41:** 255–258.

Olah I., and Glick B. (1978). Secretory cells in the medulla of the bursa of Fabricius. Experentia **34:** 1642–1643.

Olah I., Rohlich P., and Toro I. (1975). Ultrastructure of Lymphoid Organs: An Electron Microscopic Atlas.(Philadelphia: Lippincott). pp. 148–171.

Pink J.R., and Rijnbeek A.M. (1983). Monoclonal antibodies against chicken lymphocyte surface antigens. Hybridoma **2:** 287–296.

Pink J.R., Vainio O., and Rijnbeek A.-M. (1985). Clones of B lymphocytes in individual follicles of the bursa of Fabricius. Eur. J. Immunol. **15:** 83–87.

Sorvari T., Sorvari R., Ruotsalainen P., Toivanen A., and Toivanen P. (1975). Uptake of environmental antigens by the bursa of Fabricius. Nature **253:** 217–219.

Van Alten P.J., and Meuwissen H.J. (1972). Production of specific antibody by lymphocytes of the bursa of Fabricius. Science **176:** 45–47.

Weber W.T. (1982). Differences in in vivo functional capacities of avian precursor B and T cells following in vitro incubation. Adv. Exp. Med. Biol. **149:** 119–126.

Weill J.-C., Reynaud C.-A., Lassila O., and Pink J.R. (1986). Rearrangement of chicken immunoglobulin genes is not an ongoing process in the embryonic bursa of Fabricius. Proc. Natl. Acad. Sci. USA **83:** 3336–3340.

Wilson T.J., and Boyd R.L. (1990a). The ontogeny of chicken bursal stromal cells defined by monoclonal antibodies. Dev. Immunol (in press).

Wilson T.J., and Boyd R.L. (1990b). Cyclophosphamide and testosterone—induced alterations in chicken bursal stroma identified by monoclonal antibodies. Immunology (in press).

Ylickomi T.J., Isola J.J., Gasc J-M., and Tuohimaa P.J. (1987). Sexual maturation-associated and estrogen-induced progesterone receptor expression in the bursa of Fabricius. J. Immunol. **138:** 3174–3178.

